# Supplementary material for: Spatio-temporal pattern and the evolution of the distributional dynamics of county-level agricultural economic resilience in China
Source: PLoS One. 2024 May 29;19(5):e0300601. doi: 10.1371/journal.pone.0300601 (PMC11135709; doi:10.1371/journal.pone.0300601)
Supplement: Attachment — Submitted filename: Response to Reviewers.docx [file pone.0300601.s002.docx]

Dear Difang Huang Editors and Reviewers:

Thank you for your letter and for the reviewers’ comments concerning our manuscript entitled “Spatio-temporal Pattern and the evolution of the distributional dynamics of county-level agricultural economic resilience in China” (ID: PONE-D-23-33108). Those comments are all valuable and very helpful for revising and improving our paper, as well as the important guiding significance to our research. We have studied comments carefully and have made correction which we hope meet with approval, the detailed corrections are listed below:

1.SUGGESTIONS 1 FROM EDITOR: *Please ensure that your manuscript meets PLOS ONE's style requirements, including those for file naming. The PLOS ONE style templates can be found at 
https://journals.plos.org/plosone/s/file?id=wjVg/PLOSOne_formatting_sample_main_body.pdf and 
https://journals.plos.org/plosone/s/file?id=ba62/PLOSOne_formatting_sample_title_authors_affiliations.pdf*

We respond to the comments from the editor：We have revised the style of the manuscript and completed formatting changes through American Journal Experts (AJE).

2.SUGGESTIONS 2 FROM EDITOR: *We suggest you thoroughly copyedit your manuscript for language usage, spelling, and grammar. If you do not know anyone who can help you do this, you may wish to consider employing a professional scientific editing service.*

We respond to the comments from the editor：Language usage, spelling and grammar were really a huge mistake for the whole quality of our article. At the same time, we tried our best to improve the manuscript and made some changes to the manuscript. We hired American Journal Experts (AJE) to help touch up our article. We hope that the revised manuscript will be acceptable to you. Instead of listing these changes here, we have labeled the revised manuscript with the trajectory of the changes. We sincerely appreciate the dedicated work of the editors/reviewers and hope that the revisions will be recognized.

3.SUGGESTIONS 3 FROM EDITOR: *PLOS requires an ORCID iD for the corresponding author in Editorial Manager on papers submitted after December 6th, 2016. Please ensure that you have an ORCID iD and that it is validated in Editorial Manager. To do this, go to ‘Update my Information’ (in the upper left-hand corner of the main menu), and click on the Fetch/Validate link next to the ORCID field. This will take you to the ORCID site and allow you to create a new iD or authenticate a pre-existing iD in Editorial Manager. Please see the following video for instructions on linking an ORCID iD to your Editorial Manager account: https://www.youtube.com/watch?v=_xcclfuvtxQ*

We respond to the comments from the editor：Corresponding author has registered an ORCID iD account

4.SUGGESTIONS 4 FROM EDITOR: *We note that Figures 1, 2 and 4  in your submission contain map/satellite images which may be copyrighted. All PLOS content is published under the Creative Commons Attribution License (CC BY 4.0), which means that the manuscript, images, and Supporting Information files will be freely available online, and any third party is permitted to access, download, copy, distribute, and use these materials in any way, even commercially, with proper attribution. For these reasons, we cannot publish previously copyrighted maps or satellite images created using proprietary data, such as Google software (Google Maps, Street View, and Earth). For more information, see our copyright guidelines: http://journals.plos.org/plosone/s/licenses-and-copyright.*

We respond to the comments from the editor：The base maps of Figures 1, 2 and 4 in our paper were downloaded from the official website of the Ministry of Natural Resources of China, processed by Arcgis and combined with the data and model, and labeled in the paper: the base maps were not modified, and no copyright problems were noticed or found. However, the issues raised by the editors and reviewers may be serious errors that we overlooked, and we failed to solve the problem of the base figure well by consulting relevant scholars, so we replaced the data in Fig. 1 and Fig. 4 with a table 3 and table 5, and presented the modeling results of Fig. 2 in the form of a table 4 as well. We hope that the revised manuscript will be acceptable to you. We sincerely appreciate the dedicated work of the editors/reviewers and hope that the revisions will be recognized.

**Table 3. Top Ten and Bottom Ten Rankings of Agricultural Economic Resilience in China, 2000, 2010 and 2020.**

| **Ranking** | **Province** | **County** | **2000** | **Province** | **County** | **2010** | **Province** | **County** | **2010** |
| --- | --- | --- | --- | --- | --- | --- | --- | --- | --- |
| **Top ten** | **Jilin** | Nongan | 0.1279038 | **Liaoning** | Zhuang he | 0.1505686 | **Liaoning** | Zhuanghe | 0.6801182 |
|  |  | Yushu | 0.1620896 | **Jilin** | Nong an | 0.1280453 |  | Zhangwu | 0.3604184 |
|  |  | Dehui | 0.1004208 |  | Yu shu | 0.1356611 |  | Kaiyuan | 0.3416738 |
|  |  | Lishu | 0.0952098 |  | Gong zhu ling | 0.1361969 | **Shandong** | Shouguang | 0.3365598 |
|  |  | Fuyu | 0.1020133 |  | Guo Er Ross | 0.1293089 |  | Linyi | 0.2879315 |
|  | **Heilongjiang** | Shuangcheng | 0.114822 | **Heilongjiang** | Shuang cheng | 0.1291066 | **Henan** | Hua | 0.3427329 |
|  |  | Wuchang | 0.1238062 | **Jiangsu** | Dongtai | 0.13916 |  | Gushi | 0.3600621 |
|  | **Jiangsu** | Huaian | 0.0956916 | **Shandong** | Shouguang | 0.1288312 | **Hubei** | Zaoyang | 0.3617367 |
|  |  | Xinghua | 0.110905 | **Guizhou** | Qixingguan | 0.19272 |  | Jianli | 0.6743819 |
|  | **Shandong** | Pingdu | 0.0972599 |  | Bijiang | 0.14948 | **Guangdong** | Suixi | 0.3404225 |
| **...** | **...** | ... | ... | **...** | ... | ... | **...** | ... | ... |
| **Bottom ten** | **Neimenggu** | Xin Barag Right Banner | 0.0000072 | **Chongqing** | Yuzhong | 0.0000234 | **Hunan** | Furong | 0.0000034 |
|  |  | Bordered Yellow Banner | 0.0000049 | **Sichuan** | Qingyang | 0.0001134 | **Chongqing** | Yuzhong | 0.0000234 |
|  | **Liaoning** | Bayuquan | 0.0000041 |  | Wuhou | 0.0001937 | **Sichuan** | Qingyang | 0.0000245 |
|  | **Guangdong** | Chanchengqu | 0.0000032 | **Xizang** | Motuo | 0.0001989 |  | Jinniu | 0.000071 |
|  | **Guizhou** | Nanmingqu | 0 |  | Cuomei | 0.0003031 |  | Wuhou | 0.0000036 |
|  | **Xizang** | Geji | 0.0000017 |  | Cuona | 0.000263 |  | Chenghua | 0.0000427 |
|  |  | Gaize | 0.0000023 |  | Zanda | 0.000242 | **Shanxi** | Xincheng | 0.0000002 |
|  | **Shanxi** | Beilin | 0.0000014 | **Shanxi** | Xincheng | 0.0000019 |  | Beilin | 0.0000009 |
|  | **Qinghai** | Chengzhong | 0.0000105 |  | Beilin | 0.0000009 |  | Lianhu | 0.000012 |
|  |  | Maqin | 0.0000103 |  | Lianhu | 0.0000078 |  | Yanta | 0.0000026 |

**Table 4. Transfer Paths of Agricultural Economic Resilience in China.**

| Year | CentreX | CentreY | XStdDist | YstdDist | AngleZ | Area | SHAPE-  Length | SHAPE-  Area |
| --- | --- | --- | --- | --- | --- | --- | --- | --- |
| 2000 | 114.36145 | 32.85563 | 14.02449 | 9.408647 | 59.16593 | 824.873114 | 74.23893 | 412.436557 |
| 2005 | 114.26587 | 33.18466 | 14.42766 | 9.618996 | 59.87773 | 867.557731 | 76.205135 | 433.778865 |
| 2010 | 114.10624 | 33.48302 | 15.01464 | 9.937382 | 62.52389 | 932.737935 | 79.102136 | 466.368968 |
| 2015 | 114.09379 | 33.75518 | 15.62425 | 10.317143 | 64.91042 | 1007.700353 | 82.247798 | 503.850176 |
| 2020 | 114.17372 | 33.59195 | 15.31744 | 9.853877 | 60.88313 | 943.552145 | 79.910665 | 471.776072 |

**Table 5. Trends in the Evolution of Differences in Agricultural Economic Resilience in China, 2000-2020.**

| **Provinces** | **2000** | **2005** | **2010** | **2015** | **2020** | **Average** |
| --- | --- | --- | --- | --- | --- | --- |
| **Beijing** | **0.000015939** | **0.000020100** | **0.000020946** | **0.000022639** | **0.000012797** | **0.000018484** |
| **Tianjin** | **0.000050442** | **0.000086866** | **0.000101580** | **0.000112655** | **0.000141782** | **0.000098665** |
| **Hebei** | **0.000051256** | **0.000061441** | **0.000058157** | **0.000060760** | **0.000055088** | **0.000057340** |
| **Shanxi** | **0.000017815** | **0.000020294** | **0.000022862** | **0.000026293** | **0.000022326** | **0.000021918** |
| **Neimenggu** | **0.000034072** | **0.000041619** | **0.000050503** | **0.000052409** | **0.000048326** | **0.000045386** |
| **Liaoning** | **0.000098392** | **0.000082674** | **0.000098429** | **0.000124537** | **0.000176023** | **0.000116011** |
| **Jilin** | **0.000051432** | **0.000074520** | **0.000092174** | **0.000086199** | **0.000065691** | **0.000074003** |
| **Heilongjiang** | **0.000035502** | **0.000043972** | **0.000049734** | **0.000063245** | **0.000098531** | **0.000058197** |
| **Shanghai** | **0.000035064** | **0.000038069** | **0.000056656** | **0.000060527** | **0.000045858** | **0.000047235** |
| **Jiangsu** | **0.000110154** | **0.000098390** | **0.000092257** | **0.000107300** | **0.000126784** | **0.000106977** |
| **Zhejiang** | **0.000064393** | **0.000056520** | **0.000047029** | **0.000057226** | **0.000055162** | **0.000056066** |
| **Anhui** | **0.000050177** | **0.000049538** | **0.000049658** | **0.000053399** | **0.000059186** | **0.000052392** |
| **Fujian** | **0.000043841** | **0.000046488** | **0.000049384** | **0.000049917** | **0.000067281** | **0.000051382** |
| **Jiangxi** | **0.000021599** | **0.000023422** | **0.000028737** | **0.000038213** | **0.000055053** | **0.000033405** |
| **Shandong** | **0.000110397** | **0.000081675** | **0.000077352** | **0.000104293** | **0.000164155** | **0.000107574** |
| **Henan** | **0.000045897** | **0.000060618** | **0.000055297** | **0.000083747** | **0.000141693** | **0.000077450** |
| **Hubei** | **0.000084054** | **0.000055028** | **0.000063577** | **0.000080605** | **0.000146094** | **0.000085872** |
| **Hunan** | **0.000041703** | **0.000046312** | **0.000045136** | **0.000051962** | **0.000064372** | **0.000049897** |
| **Guangdong** | **0.000083694** | **0.000061969** | **0.000058895** | **0.000074310** | **0.000098332** | **0.000075440** |
| **Guangxi** | **0.000041849** | **0.000040267** | **0.000035398** | **0.000031758** | **0.000027295** | **0.000035313** |
| **Hainan** | **0.000054019** | **0.000074517** | **0.000060644** | **0.000059713** | **0.000053554** | **0.000060490** |
| **Chongqing** | **0.000031672** | **0.000040439** | **0.000042503** | **0.000053665** | **0.000085222** | **0.000050700** |
| **Sichuan** | **0.000030758** | **0.000032765** | **0.000032158** | **0.000020664** | **0.000021543** | **0.000027577** |
| **Guizhou** | **0.000070165** | **0.000020122** | **0.000065723** | **0.000018642** | **0.000027401** | **0.000040411** |
| **Yunnan** | **0.000021429** | **0.000021307** | **0.000027970** | **0.000038882** | **0.000045688** | **0.000031055** |
| **Xizang** | **0.000003835** | **0.000003846** | **0.000003225** | **0.000003490** | **0.000004283** | **0.000003736** |
| **Shanxi** | **0.000020133** | **0.000023865** | **0.000034820** | **0.000041927** | **0.000056058** | **0.000035361** |
| **Gansu** | **0.000019461** | **0.000020942** | **0.000019247** | **0.000021032** | **0.000026050** | **0.000021346** |
| **Qinghai** | **0.000017783** | **0.000015258** | **0.000014757** | **0.000013650** | **0.000012852** | **0.000014860** |
| **Ningxia** | **0.000025823** | **0.000024741** | **0.000030228** | **0.000032494** | **0.000027559** | **0.000028169** |
| **Xinjiang** | **0.000024726** | **0.000030494** | **0.000039404** | **0.000046964** | **0.000040781** | **0.000036474** |
| **Average** | **0.000045402** | **0.000045099** | **0.000049176** | **0.000054617** | **0.000066865** | **0.000450002** |
| **Eastern Region** | **0.000390287** | **0.000427606** | **0.000415092** | **0.000467204** | **0.00054982** | **0.000320934** |
| **Central Region** | **0.000261244** | **0.000255213** | **0.000265267** | **0.000334219** | **0.000488724** | **0.000370388** |
| **Western Region** | **0.000341705** | **0.000315666** | **0.000395936** | **0.000375577** | **0.000423058** | **0.000248211** |
| **Northeast Region** | **0.000185326** | **0.000201166** | **0.000240337** | **0.000273981** | **0.000340245** | **0.000018484** |

In the fourth suggestion of the editor, we failed to identify our problem with the base figure, so the copyright issue pointed out by the editor and reviewers may be a problem that we failed to identify, and once again, we are grateful to the editor and reviewers for their conscientiousness, and therefore we we had to delete Figures 1, 2, and 4, and then attach a tabular form of the relevant results (this was the first revision).

However, in a letter dated January 3, 2024, the journal asked us to attach Figures 3, 4, and 5 again, and our understanding was that the base figure could not be deleted, so we attached all of Figures 1-5 from the first submission (the second revision), and replaced the table from the first revision with Figures 1, 2, and 4 that needed to be dealt with the first time in the second revision.

It should be noted that our revision may not satisfy the editor and reviewers, but we try to understand the editor's and reviewers' opinions and make positive changes. If our second revision is incorrect, then we are happy to make the revision again until the editor and reviewers are satisfied.

5.SUGGESTIONS 5 FROM EDITOR: *Please review your reference list to ensure that it is complete and correct. If you have cited papers that have been retracted, please include the rationale for doing so in the manuscript text, or remove these references and replace them with relevant current references. Any changes to the reference list should be mentioned in the rebuttal letter that accompanies your revised manuscript. If you need to cite a retracted article, indicate the article’s retracted status in the References list and also include a citation and full reference for the retraction notice.*

We respond to the comments from the editor：We were really sorry for our careless mistakes. We sincerely thank the reviewer for careful reading, as suggested by the reviewer, We have deleted documents 30 and 47 from the original manuscript. At the same time, we have reordered the serial numbers of the references throughout the text.

ADDITIONAL EDITOR COMMENTS 1: *Improving the literature review*

1. We sincerely appreciate the valuable comments. We have checked the literature carefully and added more references on Li et al. (2023) Yu et al. (2023a) and Yu et al. (2023b) into the INTRODUCTION part in the revised manuscript, we hope that the revised manuscript will be acceptable to you. First, because COVID-19 has made significant changes in the global economy and the introduction does not present the reasons for this, the article by Li et al. (2023) needs to be cited to enhance the context of the introduction and to provide valuable information on the economic resilience of agriculture. ; second, Resilience is expressed as risk resistance to future uncertainty, and the agricultural economy, as an important part of China's economy, is very different from the financial market, but it is also of relevance. But Yu & Huang's study provides an important idea for this paper: uncertainty about future outcomes is a key driver of the behavior of economic agents, which is clearly correlated with the market, but we know little about the predictability of market excess returns in the time series. Therefore, under the uncertain outcome of a shock, the agricultural sector needs to make timely adjustments to stabilize the agricultural economy and enhance resilience to capture future market excess returns. Therefore, we have added the following literature:

Li N, Chen M, Gao H, et al. Impact of lockdown and government subsidies on rural households at early COVID-19 pandemic in China. China Agric. Econ. Rev. 2023; 15: 109-133.

Yu D, Huang D. Cross-sectional uncertainty and expected stock returns. J Empir Finance. 2023; 72: 321-340.

Yu D, Huang D. Option-Implied Idiosyncratic Skewness and Expected Returns: Mind the Long Run. Available at SSRN 4323748. 2023.

2. We sincerely thank you for your valuable comments on the literature review, and by carefully reading the total number of articles in the literature, we need to add citations to Bao & Huang, Yu et al. (2023), Chen et al. (2022), Wu et al. (2023), and Li et al. (2022) in the Literature Review section of the revised manuscript, we hope that the revised manuscript will be acceptable to you. First, although this paper does not focus on the impact of COVID-19 on the resilience of the agricultural economy, the relationship between the two cannot be ignored, and we draw on the studies of Bao & Huang (2021) and Yu et al. (2023) to point out that when China's economic growth is subjected to sudden shocks, the financial mechanism will play a positive role in it, which is very important reference to the stabilization of the agricultural economy and the enhancement of its resilience. enhancement is of great importance. Second, the development of the agricultural economy is sometimes similar to changes in the market, with certain volatility, risk, contagion and spillover. All we must draw on Chen et al. (2022) and Wu et al. (2023) studies exploring market connectivity, risk spillovers, and abnormal stock price volatility to understand the potential impacts of agricultural markets, as well as economic instability and volatility, on the resilience of the agricultural economy, which is important for promoting the high-quality development of the agricultural economy. Finally, when we study the resilience of the agricultural economy, we have to take into account that when the economy is adversely affected, the measures taken by the government as well as the resilience system established are of great significance to agriculture, and the study by Li et al. (2022) aptly describes that the adverse impacts cause many losses in agriculture, but that policy interventions in the face of external shocks can establish stable and sustainable agricultural supply chains to ensure that rural households are able to survive during an epidemic viability during an epidemic, which provides valuable information on the impact of economic resilience in agriculture. Therefore, it is possible to better illustrate the importance of studying the resilience of the agricultural economy and its significance for the formulation of sound and effective policies and the promotion of high-quality development of the agricultural economy. Therefore, we add the following literature:

Bao Z, Huang D. Shadow banking in a crisis: Evidence from FinTech during COVID-19. J. Financial Quant. Anal. 2021; 56: 2320-2355.

Yu D, Huang D, Chen L. Stock return predictability and cyclical movements in valuation ratios. J Empir Finance. 2023; 72: 36-53.

Chen M, Li N, Zheng L, et al. Dynamic correlation of market connectivity, risk spillover and abnormal volatility in stock price. Physica A. 2022; 587: 126506.

Wu B, Huang D, Chen M. Estimating contagion mechanism in global equity market with time‐zone effect. Financ Manag., 2023; 52: 543-572.

Li N, Chen M, Huang D. How Do Logistics Disruptions Affect Rural Households? Evidence from COVID-19 in China. Sustainability. 2022; 15: 465.

ADDITIONAL EDITOR COMMENTS 2: *Detailed comments to improve the submission*

1. *For a more detailed explanation of the methodology used to calculate the agricultural economic resilience index*. In the article, we have explained the methodology for calculating the agricultural economic resilience index (entropy weight method) in more detail, focusing on the standardization of indicators, standardization of definitions, and determination of information entropy value and information utility value of the indicators, in the hope that it will help readers to better understand the process and principles behind the chosen methodology, and we hope that the revised manuscript will be acceptable to you.

2. *For discussing the potential limitations*. We have added potential limitations of the article's study at P36-37, Line 613-634 as suggested by the editor and reviewers. The three main aspects of the agricultural economic resilience indicator system needs to be expanded, the driving factors of agricultural economic resilience need to be screened and mechanism of inter-regional agricultural resilience and the internal and external environments need to be studied in depth, and we have taken these limitations as the content of our next research. at the same time, we hope that the revised manuscript will be acceptable to you.

3. *Include a discussion on the policy implications of your findings*. We think this is an excellent suggestion, We have the change made in the revised manuscript. First, the results of the study found that the difference between the agricultural economic resilience of some counties and that of large agricultural provinces is large, mainly due to the low level of agricultural technology and the relative lack of scientific and technological personnel, which impede the improvement of the resilience level of the agricultural economy. Agricultural production services can promote the low toughness of the region to promote scientific and technological innovation, drive the level of agricultural mechanization, through the "State Council's Guiding Opinions on Accelerating the Development of the Productive Service Industry to Promote the Adjustment and Upgrading of the Industrial Structure (Guo Fa [2014] No. 26)", can better promote the transformation and upgrading of the agricultural industrial structure in order to enhance the agricultural economic toughness of the agricultural economic toughness of the region's agricultural risk-resistant ability. Second, the results of the study found that the spatial effects of agricultural economic resilience in China's counties are continuous and affect each other. Therefore, based on the regional spatial distribution pattern of agricultural economy, we divide the city clusters with high or concentrated levels of agricultural economic resilience, and draw on the effectiveness of the implementation of policies such as the Yangtze River Delta Regional Ecological Environment Protection Plan, the Action Plan for the Continuous Improvement of Air Quality, and the Chengdu-Chongqing Twin Cities Economic Circle Construction Plan, to construct a mechanism for regional collaboration on agricultural economic resilience, so as to enhance the agricultural economic resilience of the regions with lower levels of agricultural economic resilience. Finally, the lower level of agricultural economic resilience in the central and western provinces may be due to the insufficient construction of agricultural basic capacity and supporting conditions in these areas, so it is necessary to strengthen financial support for counties within the central and western provinces. It is possible to set up special earmarked funding projects through the policy advice of the "Guidance on Financial Support for Comprehensively Promoting Rural Revitalization and Accelerating the Construction of a Strong Agricultural Country" to enhance the incremental amount of agricultural capital and other livelihood funds, which will in turn enhance the agricultural risk-resistant capacity of the areas in the central and western regions where the agricultural economic resilience is low.

We look forward to hearing from you again.

Kind regards,

Guoxin Yu, Prof.
