# Supplementary material for: Spatio-temporal pattern and the evolution of the distributional dynamics of county-level agricultural economic resilience in China
Source: PLoS One. 2024 May 29;19(5):e0300601. doi: 10.1371/journal.pone.0300601 (PMC11135709; doi:10.1371/journal.pone.0300601)
Supplement: Attachment — Submitted filename: Response to Reviewer.docx [file pone.0300601.s003.docx]

Dear Editor and Reviewer:

Thank you for your letter and for the reviewers’ comments concerning our manuscript entitled “Spatio-temporal pattern and the evolution of the distributional dynamics of county-level agricultural economic resilience in China” (ID: PONE-D-23-33108R1). Those comments are all valuable and very helpful for revising and improving our paper, as well as the important guiding significance to our research. We have studied comments carefully and have made correction which we hope meet with approval, the detailed corrections are listed below:

1.SUGGESTIONS 1 FROM REVIEWER 1: *Please indicate the map review number for the Chinese map in the article, and ensure consistency in elements like the legend and compass orientation.*

We respond to the comments from the reviewer 1：Thank you very much for your suggestion. First of all, for the issue of review number, we have replaced "Review No." with "Map Review Number for the Chinese:" under Figs. 1, 2, 4, etc., in which GS (2022) 1873 is the map review number. Secondly, regarding the compasses and legends of Figs. 1 and 4, we have improved the compass of Fig. 2. Moreover, in our article, the legends of Figures 1 and 4 are consistent with the compass, whereas the legend of Figure 2 is somewhat different because of what it represents. Therefore, we changed the compass and did not modify the legend. Finally, if there are still problems with our charts, we will continue to improve them. Thank you for your valuable comments and we hope that our changes will satisfy you.

2.SUGGESTIONS 2 FROM REVIEWER 1: *Tables should be formatted using the three-line table format.*

We respond to the comments from the reviewer 1：We are very sorry that the formatting error led to such problems in our article. According to the reviewer's suggestion, we have revised Table 1 and 2 to three-line table format, and we are very grateful to the reviewer for his valuable comments, and we hope that our revisions will be satisfactory to you.

3.SUGGESTIONS 3 FROM REVIEWER 1: *For the discussion section, consider organizing paragraphs to present one point per paragraph for a more aesthetically pleasing layout.*

We respond to the comments from the reviewer 1：Thank you very much for your suggestions and giving us the opportunity to improve the article. We have added three sub-chapters to the “Discussions” section of the article: conclusions, policy recommendations, limitations and future recommendations. We hope that our revisions will satisfy you.

4.SUGGESTIONS 4 FROM REVIEWER 1: *While the introduction introduces the research background, there is limited mention of the summary and shortcomings of past research. Additionally, the innovative aspects and practical significance of this study are not clearly emphasized. It is recommended to supplement this information.*

We respond to the comments from the reviewer 1：The reviewer's suggestion was very relevant, pointing out that our article was missing a part of the content. We explained that we have included the summary of past studies and their shortcomings in the second part of the article (Literature Review). We also point out that most of the spatial mechanism studies in the literature are limited to regional "proximity" or "similarity", but lack the overall perspective of correlation, and are mostly based on "relationship" rather than "quantity". We have not yet considered the correlation from a holistic perspective, and we have mostly studied the spatial and temporal patterns of agricultural economic resilience from the perspective of "relationship" rather than "quantity". Meanwhile, in the last paragraph, we propose three possible contributions or innovations of this study. We hope that our explanations will satisfy the reviewers, and we thank them for their suggestions.

6.SUGGESTIONS 1 FROM REVIEWER 2: *The article should first write about the construction of the indicator system, and then write about the research methods.*

We respond to the comments from the reviewer 2：We would like to thank the reviewers for their valuable comments, which helped us to improve our article. According to the reviewer's suggestion, we have moved "construction of the indicator system" to the front of "research methods". We hope that you will be satisfied with our revision.

7.SUGGESTIONS 2 FROM REVIEWER 2: *The research methods are all mature and there is no need to introduce them in such detail.*

We respond to the comments from the reviewer 2：The suggestions made by the reviewers were very reasonable. Since the reviewers suggested a detailed explanation of the methodology for measuring the economic resilience of agriculture during the first round of review, we have introduced the entropy right method extensively. Therefore, we are also very grateful for this valuable advice and have abridged the introduction of the entropy right method. As for the other research methods, we think there is no main content missing and a short introduction has been made, so no addition or deletion of content has been made, and we have only revised and improved the entropy right method. We thank the reviewers for their suggestions, and hope that our revision will satisfy you.

8.SUGGESTIONS 3 FROM REVIEWER 2: *There are certain problems with the indicator system, such as the lack of necessary connection between rural electricity consumption and innovation capacity.*

We respond to the comments from the reviewer 2：Many thanks to the reviewers for pointing out the problems with the indicators in our article, such as the lack of the necessary link between rural electricity consumption and innovation capacity. Here we make explanations:

1. electricity is an important driver of innovation: electricity is the basis of modern agriculture and scientific and technological development, and it provides the necessary energy for innovative activities. With a stable supply of electricity, all kinds of innovative activities in rural areas can be carried out smoothly, from the research and development of agricultural technology to the operation of rural enterprises, all of which need the support of electricity.

2. Rural electricity consumption reflects the level of economic development: Generally speaking, the electricity consumption of a region can be used as an important indicator to measure its level of economic development. Higher electricity consumption usually means that there is more business and industrial activity in the area, which provides the necessary economic base and market environment for innovation.

3. The relationship between electricity consumption and education, training and access to information, e.g.: in rural areas, an increase in the availability of electricity may also mean an increase in education and training opportunities. More electricity means more possibilities to use modern technological means (e.g. the Internet) to access knowledge and information, which is crucial for fostering a sense of creativity and enhancing innovation.

4. Electricity consumption has an important relationship with technology adoption and innovation: an increase in rural electricity consumption is likely to promote the adoption and diffusion of agricultural technologies, including more efficient irrigation systems, agricultural mechanization, etc. This not only directly enhances agricultural production, but also increases the availability of agricultural technologies. This not only directly improves the efficiency of agricultural production, but also provides the necessary material basis for agricultural innovation.

5. Rural electricity consumption is related to infrastructure development: Government investment in infrastructure in rural areas, including the construction of electricity networks, may directly contribute to rural economic development and innovative activities. For example, by providing electricity, the government may create a more innovation-friendly environment in rural areas.

In summary, rural electricity consumption not only directly reflects the level of economic development and commercial and industrial activity in the region, but also indirectly affects innovation capacity by influencing education and access to information, technology adoption, and the building of an innovative environment. Therefore, rural electricity consumption is an important reference point when assessing the indicator of reconfigured innovation capacity.

Finally, we would like to thank the reviewers for their valuable comments and hope that our explanations will satisfy you.

9.SUGGESTIONS 4 FROM REVIEWER 2: *The sample range is said to be 31 provinces, but there are no 31 provinces in Table 2.*

We respond to the comments from the reviewer 2：Many thanks to the reviewers for their suggestions, we rechecked the contents of Table 2 and determined that it was a range that included 31 provinces. It is possible that we did not have a three-line table, which caused the table to be unclear, so we have changed the form of the table to a three-line table. We hope that our explanation is satisfactory to you.

10.SUGGESTIONS 5 FROM REVIEWER 2: *The paper lacks innovation.*

We respond to the comments from the reviewer 2：Many thanks to the reviewers for pointing out the lack of innovation in our article. In this regard, we have done the following two aspects to improve the innovation: on the one hand, most scholars' research scale is only centered on provincial and prefecture-level city units [39-47], but our innovation in this study is to take 2,545 county units as the scope of the study, which improves the innovation of the study in terms of the research scale. On the other hand, we apply the methods of "three-stage nested Theil index" and "stochastic Kernel density estimation" to the spatial differences and dynamic distribution of agricultural economic resilience, which can serve as a reference for related studies. Moreover, these two methods have certain novelty in themselves. To sum up, although there are many existing studies on the toughness of agricultural economy, there is still a big difference with our study, and the purpose of this study is to analyze the shortcomings of China's agricultural economic development, in order to build a strong agricultural country, and to improve the toughness of agricultural economy in the lower counties, which will help to improve China's agricultural economy as a whole. Finally, we are very grateful to the reviewers for pointing out the problems, and we hope that our explanations will satisfy the reviewers. Meanwhile, we are already studying the mechanism of digital finance, production services, digital economy and other elements on the resilience of the agricultural economy, which is used to make up for the current shortcomings in front of us.

11.Journal Requirements: *Please review your reference list to ensure that it is complete and correct. If you have cited papers that have been retracted, please include the rationale for doing so in the manuscript text, or remove these references and replace them with relevant current references. Any changes to the reference list should be mentioned in the rebuttal letter that accompanies your revised manuscript. If you need to cite a retracted article, indicate the article’s retracted status in the References list and also include a citation and full reference for the retraction notice.*

We respond to the comments from the Journal Requirements：We checked the references again on the basis of the previous round of revision. At the same time, we found some problems, such as citing CNKI, books, poor quality, unclear retrieval and so on. Therefore, we replaced references 10, 20, 41, 45, 49 and 51 so that journals can retrieve them and check the retracted articles to correct our wrong behavior.

Thank you again.

Kind regards,

Guoxin Yu, (Prof. )
